# Mediterranean Diet, Physical Activity, and Sleep in Endometriosis: An Age-Comparable Case-Control Study with Exploratory Machine Learning Discrimination

**DOI:** 10.3390/nu18213629

**Published:** 2026-10-31

**Authors:** Azahara Ruger Navarrete, Miguel Ángel Avellaneda Roldán, Naore López Gómez, Luciano Díaz Rodríguez, Concepción Roldán, Beatriz Mérida Yáñez

**Affiliations:** 1 Department of Nursing, Faculty of Health Sciences, Campus Universitario de Ceuta, University of Granada, 51001 Ceuta, Spain; azahara.ruger@ugr.es (A.R.N.); luciano.diaz@go.ugr.es (L.D.R.); bmerida@ugr.es (B.M.Y.); 2 Department of Statistics and Operational Research, Faculty of Health Sciences, Campus Universitario de Ceuta, University of Granada, 51001 Ceuta, Spain; naorelg@go.ugr.es (N.L.G.); iroldan@ugr.es (C.R.)

**Keywords:** endometriosis, Mediterranean diet, physical activity, sleep, quality of life, case-control study, machine learning

## Abstract

Background/Objectives: Diet, physical activity, and sleep have been proposed as modulators of endometriosis, yet most evidence comes from studies in which cases are much older than controls, confounding lifestyle comparisons by age. We compared these profiles against controls of closer reproductive age and, as a secondary aim, explored how far self-reported data discriminate endometriosis. Methods: Cross-sectional case-control study of 177 women: 77 with self-reported, procedure-confirmed endometriosis recruited through a patient association and 100 controls consecutively enrolled from the general adult population. Measures included Mediterranean diet adherence (PREDIMED-14), physical activity (IPAQ), pain scales, menstrual quality of life (CVM-22) and the Epworth Sleepiness Scale. Analyses used age-adjusted logistic regression with Benjamini–Hochberg correction; three machine learning algorithms were evaluated with cross-validation. Results: Mediterranean diet adherence and physical activity did not differ between groups; body mass index was modestly lower in cases, and daytime sleepiness was marginally higher but not after age adjustment. Of the 13 testable dietary items, only sofrito (vegetables slowly cooked in extra-virgin olive oil) survived correction, being reported less often by cases. Menstrual quality of life was markedly worse in cases. Discrimination (best AUC-ROC 0.933) reflected concurrent symptoms rather than nutrition: restricted to antecedent and dietary variables it reached only 0.67–0.73. Conclusions: Once age and multiple testing were accounted for, diet and physical activity did not distinguish women with endometriosis from controls. The lower sofrito intake is a single-item, cross-sectional finding that should be regarded as hypothesis-generating and requires independent validation.

## 1. Introduction

Endometriosis is a chronic, systemic, inflammatory, and estrogen-dependent condition defined by the presence of endometrial-like tissue outside the uterine cavity [1]. It affects approximately 10% of women of reproductive age worldwide, with an estimated global prevalence of 190 million cases [2]. The condition is characterised by dysmenorrhoea, chronic pelvic pain, dyspareunia, and infertility, profoundly affecting physical, psychological, and social well-being [3].

Despite its high prevalence, the average time from symptom onset to definitive diagnosis ranges from 7 to 10 years [4,5]. This protracted diagnostic journey generates a prolonged period of ‘biological noise’, characterised by progressive—often irreversible—deterioration of quality of life while non-specific symptoms are misattributed to other conditions such as irritable bowel syndrome and primary dysmenorrhoea, a symptom overlap that is central to the diagnostic difficulty [5,6,7]. The continued reliance on laparoscopy as the gold standard for confirmation compounds this problem and has motivated interest in non-invasive tools grounded in standardised self-report of multidimensional clinical phenotypes. This line of work has been pursued by major European consortia; the EU-funded FEMaLe (Finding Endometriosis using Machine Learning) project showed that self-tracked patient data can feed machine learning models with useful diagnostic accuracy [8].

Beyond pain-related clinical symptoms, lifestyle factors—particularly dietary patterns, physical activity, and sleep quality—have been proposed as modulators of the inflammatory pathways underlying endometriosis [9]. Adherence to the Mediterranean diet has been associated with reduced menstrual pain, including in Spanish student populations [10], and, in one case-control study, with up to a 94% reduction in the odds of endometriosis (OR = 0.06; 95% CI: 0.02–0.17) [11]; a further case-control study reported that adherence to a fertility-oriented dietary pattern approximately halved the odds of the disease [12]. Effects of this magnitude should, however, be interpreted cautiously, as such estimates from case-control designs are susceptible to residual confounding (notably by age) and reverse causation—a concern that directly motivated the age-comparable design of the present study. The proposed mechanistic basis is the downregulation of pro-inflammatory cytokines (IL-1β, IL-6, TNF-α) by polyunsaturated omega-3 fatty acids, natural antioxidants, and dietary fibre, which may promote intestinal microbiota homeostasis and oestrogen clearance [13]. Consistent with an effect at the level of the target tissue, adherence to the Mediterranean diet has been associated with the whole-metabolome profile of the endometrium during the receptive phase [14]. Women with endometriosis, however, frequently report suboptimal dietary quality and limited nutritional knowledge [15]. The overall evidence is not unanimous: a 2026 Clinical Nutrition ESPEN review concluded that, although global dietary patterns such as the Mediterranean diet may offer indirect benefits on systemic inflammation and overall well-being, current evidence does not support any specific diet as a disease-specific treatment for endometriosis, and restrictive ‘anti-inflammatory’ diets remain insufficiently substantiated by high-quality trials [16]. The Mediterranean diet also involves how foods are cooked, not only which foods are eaten. Sofrito—onion, garlic and tomato slowly cooked in extra-virgin olive oil, the base of many traditional Spanish dishes—is the only PREDIMED-14 item that reflects culinary technique rather than food purchase; this preparation has been reported to increase the extractability of polyphenols and carotenoids [17] and the formation of cis isomers of lycopene [18]. Physical inactivity amplifies systemic oxidative stress and peripheral aromatisation of oestrogens, and has been associated with greater endometriosis symptom severity [13,19]. Sleep disturbances are also reported in endometriosis, reflecting both pain-driven circadian disruption and potentially independent neuroinflammatory mechanisms [20]. Evidence on the comparative lifestyle and dietary profile of women with and without endometriosis remains limited, particularly in culturally diverse Southern European populations.

Machine learning (ML) algorithms have demonstrated considerable potential in gynaecological diagnostics by identifying complex, non-linear patterns in clinical data [21]. Previous studies have used biomarkers, imaging features, and symptom-based variables for non-invasive endometriosis prediction [22,23]. Konrad et al. (2023) reported that clinical parameters alone can reach AUC values of approximately 0.80 [23]. However, most published ML models do not simultaneously integrate validated quality-of-life instruments alongside dietary and lifestyle variables, and none have been validated in culturally diverse Spanish cohorts.

The primary objective of this study was therefore to characterise and compare lifestyle, dietary, and sleep profiles between women with and without endometriosis using validated instruments (PREDIMED-14, IPAQ, ESS), with explicit control for age as a confounder and for multiple comparisons. A secondary, exploratory objective was to quantify how far exclusively self-reported variables—pain intensity (VAS), quality of life (CVM-22), daytime sleepiness (ESS), Mediterranean diet adherence, physical activity (IPAQ), and sociodemographic characteristics—discriminate women with diagnosed endometriosis from controls, and in particular how much of any such discrimination is attributable to nutrition and lifestyle rather than to established symptoms. Given the cross-sectional, case-control design, the discrimination analysis is a proof-of-concept and not a validated risk prediction or screening tool.

## 2. Materials and Methods

### 2.1. Study Design and Ethical Considerations

This was a cross-sectional, case-control observational study. Ethical approval was obtained from the Ethics Committee for Research with Human Beings of the University of Granada (CEIH-UGR; protocol code 5708/CEIH/2026; approval date: June 2026). All participants provided digital informed consent via the LimeSurvey platform (LimeSurvey GmbH, Hamburg, Germany) prior to accessing the questionnaire, in accordance with the Declaration of Helsinki and the General Data Protection Regulation (EU 2016/679). The protocol was submitted to the committee in February 2026 and approved in June 2026, the interval reflecting several rounds of requested clarifications. The questionnaire had been prepared in advance and was therefore launched immediately after approval, with data collection taking place during June 2026.

Responses were pseudonymised rather than fully anonymised: each participant entered a personal code derived from several digits of her national identity document, known only to herself, so that she could identify her own record without being identifiable to the research team or to other participants. The pseudonymisation procedure was covered by the approved protocol and by the informed consent.

### 2.2. Participants, Recruitment and Reference Standard

The case-control sample comprised 177 women: 77 with endometriosis (endometriosis group) and 100 age-comparable controls. The endometriosis group was recruited through the national patient association ADAEC (Asociación de Afectadas de Endometriosis Crónica), requiring a diagnosis previously confirmed by laparoscopy or advanced imaging (transvaginal ultrasound or MRI), consistent with the diagnostic standards of the ESHRE endometriosis guideline [24]. It must be emphasised that diagnostic status, the confirmatory procedure and the ASRM stage were reported by the participants themselves and were not verified against clinical or surgical records, which were not accessed because the survey was pseudonymised at source; misclassification of case status or stage therefore cannot be excluded (Section 4.1). To reduce this risk, participants were required to specify which confirmatory procedure they had undergone rather than simply to affirm a diagnosis, and membership of a patient association typically follows a prolonged diagnostic process. To avoid subjective selection bias by the researchers, controls were enrolled by strict consecutive sampling from the general adult population of the authors’ catchment area in southern Spain (Andalusia and Ceuta), approached through community settings, until 100 age-comparable participants were reached (their geographic distribution is reported in Section 3.1 and Figure S1). Women with suspected but unconfirmed endometriosis, and those who were unsure of their status, were explicitly excluded. Data were collected through a structured self-administered questionnaire administered via LimeSurvey, a secure server hosted by the University of Granada, guaranteeing confidentiality.

Inclusion criteria were: female sex, age 18–55 years, and voluntary written informed consent. Exclusion criteria for the control group included any prior medical suspicion or confirmed diagnosis of endometriosis and uncertainty about one’s status.

### 2.3. Study Variables

The questionnaire comprised eight domains: (1) sociodemographic and anthropometric variables (age, weight, height and waist circumference—from which body mass index [BMI] was derived—smoking, and geographic origin/cultural identification); (2) physical activity (International Physical Activity Questionnaire—IPAQ, short version; classified as low/moderate/high); (3) Mediterranean diet adherence (14-item PREDIMED questionnaire, range 0–14; adherence ≥ 8 considered adequate [25]; a brief adherence screener of largely dichotomous items that does not quantify food quantities, nutrient intakes or dietary patterns beyond its fourteen items); household income (three categories: below EUR 20,000, EUR 20,000–40,000, and above EUR 40,000 per year) and religious or cultural identification were also recorded, the former being used to test socioeconomic confounding of the dietary comparisons; (4) gynaecological and clinical data (confirmed diagnosis, ASRM disease stage, surgical history—recorded as a free-text description of any previous surgery and categorised for this analysis as laparoscopic excision, hysterectomy, laparotomy, bowel resection or other gynaecological procedure—first-degree family history, age at menarche, menstrual cycle regularity); (5) pain intensity by Visual Analogue Scale (VAS, 0–10) for dysmenorrhoea, dyspareunia, chronic pelvic pain, and pain with urination/defecation, together with four items on pain-related functional impairment (persistent daily pelvic pain, interruption of tasks requiring standing, altered walking or mobility, and need for bed rest or cancelled plans), each rated on a 5-point frequency scale (0 = never to 4 = almost always); (6) menstrual-related quality of life assessed with the validated CVM-22 (Calidad de Vida relacionada con la Menstruación) scale; 22 items, each scored on the instrument’s 4-point frequency scale (0 = not at all to 3 = very frequent), giving a total score of 0–66 (higher = worse menstrual-related quality of life) [26]; item labels in the results tables are abbreviated summaries of the questionnaire items. One item (item 19, asking whether pain subsides with the usual analgesic treatment) is worded so that a higher score denotes a better outcome, unlike the remaining twenty-one; we therefore repeated the total-score comparison excluding this item and with it reverse-scored, and the group difference was unchanged in either case (Section 3.2); (7) daytime sleepiness assessed with the validated Epworth Sleepiness Scale (ESS, 8 items, 0–3 per item; total 0–24; scores ≥ 10 indicate excessive daytime sleepiness) [27]; (8) hormonal contraceptive use (type and duration of use).

### 2.4. Machine Learning Pipeline

The sample comprised all eligible women recruited through the participating patient association together with the consecutive enrolment of controls, rather than a prospectively calculated target. Following current recommendations for the development of clinical prediction models [28], the analysis is therefore framed as exploratory and proof-of-concept: we relied on penalisation and repeated cross-validation to limit overfitting, and we report cross-validated alongside test-set performance so that any optimism is visible (Section 3.3 and Section 4.1). The dataset was split 70:30 into training (*n* = 123) and test (*n* = 54) sets by stratified random sampling. Three algorithms were compared—Elastic Net logistic regression, Random Forest, and XGBoost—with hyperparameters tuned by 10-fold repeated cross-validation (5 repeats) and class imbalance addressed with SMOTE applied only to the training data [29,30]. The candidate predictor set comprised 54 variables in the full model and 45 in the model without pain; regularisation and ensemble averaging were relied upon to limit overfitting, which is discussed further in Section 4.1. Full pipeline specifications—resampling seeds, imputation of the few missing values (<5% for all predictors), SMOTE settings and tuning grids—are given in Supplementary Methods S1. All analyses used R 4.6.0 (R Foundation for Statistical Computing, Vienna, Austria) and the tidymodels framework (version 1.5.0) [31].

### 2.5. Statistical Analysis and Model Evaluation

Descriptive group comparisons used Student’s *t*-test for approximately symmetric continuous variables (reported as mean ± SD), the Mann–Whitney U test for ordinal or skewed variables (VAS, CVM-22 and ESS items, and the CVM-22 total score, whose distribution in cases was bimodal; reported as median [IQR]), and chi-squared or Fisher’s exact test for categorical variables (alpha = 0.05). Because a residual age difference persisted between cases and controls, the principal dietary, physical activity, and sleep comparisons were additionally assessed with age-adjusted binary logistic regression (endometriosis status regressed on each factor plus age), reporting adjusted odds ratios and *p*-values. To distinguish risk factor association from the discrimination model, a separate logistic regression was fitted using only antecedent (pre-disease) variables—first-degree family history, age at menarche, body mass index, and smoking—both unadjusted and adjusted for age; symptom, pain, quality-of-life, and sleep variables were excluded from this model because they are consequences rather than antecedents of the disease. To control the false discovery rate arising from the large number of item-level tests, Benjamini–Hochberg-adjusted q-values were computed separately within each instrument family (the 13 testable PREDIMED items—the olive oil item was met by every participant and therefore carries no between-group variance—22 CVM-22 items, and 8 ESS items); item-level findings are considered robust only when q < 0.05. Sensitivity analyses examined whether the dietary findings depended on previous surgery (excluding women with bowel resection, whose post-operative bowel function could affect pain and quality of life) and whether symptom severity modified them (age-adjusted odds ratios estimated separately for cases above and below the median of constant pelvic pain and of the CVM-22 total score, each against all controls). Model performance on the held-out test set was summarised by discrimination (AUC-ROC and AUC-PR, with bootstrap 95% confidence intervals), the usual threshold-based measures at a 0.5 cut-off, and calibration (Section 3.3). To guard against overfitting optimism, the cross-validated AUC-ROC is reported alongside the held-out value. Variable importance for Random Forest was estimated by mean decrease in Gini impurity; because this measure can favour continuous and high-cardinality predictors, it is interpreted qualitatively and read together with the univariate group comparisons (Section 3.2) and the standardized Elastic Net coefficients (Supplementary Table S1). Finally, to test whether discrimination depended on the size of the feature set, we fitted a parsimonious ordinary (unpenalised) logistic model whose number of predictors respects the sample-size limit for prediction models [28], using the same data split and cross-validation scheme as the main models; its predictors were chosen from the highest-ranked, mutually non-collinear variables.

## 3. Results

### 3.1. Participant Characteristics

The main case-control sample comprised 177 women: 77 with endometriosis (mean age 37.7 ± 7.6 years) and 100 age-comparable controls (mean age 31.7 ± 8.2 years). Recruiting controls of comparable reproductive age substantially narrowed the age gap typical of this literature, although a residual difference of about six years persisted and cases remained older (*p* < 0.001); age-adjusted models were therefore used throughout. Table 1 presents baseline clinical characteristics. A self-reported ASRM stage was available for all 77 patients: 32 reported stage IV, 11 stage III, 16 stage II and 18 stage I, so that advanced disease (stages III–IV, 43 women, 56%) formed the largest share. Nineteen patients were receiving endometriosis-specific hormonal therapy and five a GnRH analogue or antagonist, treatments that no control participant reported. The endometriosis group reported substantially higher dyspareunia, constant pelvic pain and pain on urination or defecation (all *p* < 0.001); dysmenorrhoea, by contrast, did not differ significantly (median 6.2 vs. 4.0; *p* = 0.098), plausibly because many patients were amenorrhoeic under hormonal suppression. First-degree family history of endometriosis was significantly more frequent in cases (*p* < 0.001). Body mass index was modestly lower in cases (endometriosis 23.7 ± 4.7 vs. control 25.1 ± 4.6 kg/m^2^; *p* = 0.049). Even against this age-comparable control group, menstrual-related quality of life was markedly worse in cases (median CVM-22 total 57 vs. 39; *p* = 0.006), and 9 of the 22 CVM-22 items remained significantly different after false discovery rate correction (two of them at the margin, q = 0.0500; Section 3.2). The distribution of the total score in cases was bimodal—patients under effective hormonal suppression clustered at low scores—so that means (42.6 vs. 38.0) understate the difference, and median-based tests are reported.

Of the 41 patients with previous surgery, 20 had undergone laparoscopic excision, 9 hysterectomy, 4 an unspecified open laparotomy, 2 bowel resection (rectosigmoid resection or hemicolectomy), and 6 other gynecological procedures (Table 1); 10 controls reported a previous procedure, none of them bowel surgery. The effect of bowel surgery for deep endometriosis on defecatory function depends on the technique: in a prospective manometric study, conservative shaving of rectosigmoid nodules preserved intestinal neurological function and improved defecatory symptoms [32], whereas both of our cases had undergone segmental procedures. We therefore repeated the main comparisons excluding the two women with bowel resection (*n* = 75 cases): pain on urination or defecation (*p* < 0.001), constant pelvic pain (*p* < 0.001), and CVM-22 total (*p* = 0.002) still differed, and the age-adjusted odds ratio for sofrito was unchanged (0.27 vs. 0.28 in the full sample). Within the endometriosis group, operated and non-operated women did not differ in pain on urination or defecation (*p* = 0.713) or constant pelvic pain (*p* = 0.242); operated women reported a lower CVM-22 total (median 48 vs. 58; *p* = 0.016), and sofrito intake did not differ by surgical history (*p* = 0.638).

Geographic origin differed markedly between groups (Fisher’s exact test *p* < 0.001; Figure S1). Cases were geographically dispersed across Spain—51.9% from Andalusia, 40.3% from the rest of Spain, 1.3% from Ceuta, and 6.5% from abroad—consistent with their recruitment through a national patient association. Controls were predominantly local/regional (75.0% Andalusia, 11.0% Ceuta, 11.0% rest of Spain, 3.0% abroad). This difference reflects the recruitment design rather than a geographic risk factor, and reinforces the selection asymmetry discussed in the Limitations; it also indicates that the sample, although geographically varied, is predominantly Spanish and cannot support claims of transcultural validity.

### 3.2. Lifestyle, Dietary and Sleep Characteristics

Table 2 presents lifestyle, dietary, and sleep comparisons between the age-comparable groups. Physical activity level showed a non-significant trend toward lower activity in cases (38% of women with endometriosis reported low or inactive activity vs. 29% of controls; 8% vs. 19% reached high activity; Fisher’s exact test *p* = 0.082; age-adjusted *p* = 0.29). Overall Mediterranean diet adherence was also comparable: PREDIMED-14 total scores did not differ (8.6 ± 2.2 vs. 8.5 ± 2.0; *p* = 0.82), and the proportion meeting the adequate adherence threshold (score ≥ 8) was virtually identical (70.1% vs. 70.0%; *p* = 0.99). Daytime sleepiness was marginally higher in cases (ESS 10.4 ± 5.6 vs. 8.8 ± 4.6; *p* = 0.039; 58% vs. 45% scored ≥10), but the difference was not significant after adjustment for age (*p* = 0.062).

At the individual PREDIMED item level, after Benjamini–Hochberg correction across the 13 testable items (the olive oil item was met by every participant), only sofrito consumption (Q14; the traditional olive oil-based vegetable preparation described in the Introduction) remained significant, being higher in controls (q = 0.025). Fish (*p* = 0.027, q = 0.166) and nut (*p* = 0.038, q = 0.166) consumption were nominally higher in cases but did not remain significant after correction. After adjusting for age with logistic regression, the fish association strengthened slightly and reached significance (adjusted *p* = 0.016; adjusted OR = 2.23), whereas nut consumption did not (adjusted *p* = 0.176). The lower sofrito consumption among cases remained significant after age adjustment (adjusted *p* = 0.001; adjusted OR = 0.28). Given that only one of fourteen items survived multiplicity correction, these item-level signals are best regarded as hypothesis-generating rather than evidence of a protective or harmful dietary pattern. Notably, the three items showing any group difference point in a consistent direction: cases reported less sofrito (62.3% vs. 83.0%) but more fish (59.7% vs. 43.0%) and more nuts (63.6% vs. 48.0%) than controls.

To characterise what sofrito consumption represents, we compared women who met its criterion with those who did not. Sofrito consumers had higher overall PREDIMED-14 adherence (median 9.0 vs. 7.5; *p* < 0.001), yet no single other dietary item differed significantly between them (for example, fruit 44% vs. 35%, legumes 46% vs. 37%, and extra-virgin olive oil as the main fat 74% vs. 65%; all *p* > 0.2). Sofrito therefore behaves less as a proxy for any one accompanying food than as a marker of an overall way of eating. Consistent with the group comparison above, sofrito consumers reported less fish (47% vs. 59%), so sofrito and fish tended to substitute for one another—the pattern of less traditional cooking alongside more individually selected ‘healthy’ foods that we return to in the Discussion.

We further examined whether the lower sofrito consumption among patients might itself be a consequence of symptom burden—for instance, if pain or fatigue limited the capacity for time-consuming cooking. Within the endometriosis group, meeting the sofrito criterion was unrelated to pain on urination or defecation (median VAS 5.0 vs. 2.0; *p* = 0.23), to dysmenorrhoea (7.0 vs. 4.0; *p* = 0.56), to total CVM-22 score (57.0 vs. 55.0; *p* = 0.82) or to age (*p* = 0.76); moreover, the non-significant differences ran in the opposite direction to that hypothesis. There was therefore no dose-response relationship between disease burden and traditional cooking within the patient group. Nor did symptom severity modify the case-control association: the age-adjusted odds ratio for sofrito was similar for cases below and above the median of constant pelvic pain (0.23, 95% CI 0.09–0.57, vs. 0.35, 0.14–0.86) and of the CVM-22 total score (0.23, 0.09–0.58, vs. 0.33, 0.13–0.80), with widely overlapping confidence intervals.

The sofrito association proved robust to every measured determinant of diet quality. Household income did not differ between groups (*p* = 0.10), sofrito intake was similar across the three income bands (75%, 71% and 79% from the lowest to the highest band), and the association was essentially unchanged after adjustment for income (adjusted OR 0.27); it was attenuated only slightly by religious or cultural identification (which did differ, *p* = 0.009, most plausibly reflecting the different recruitment routes; adjusted OR 0.32), remaining significant with age, income and identification together (OR 0.30; *p* = 0.003); only three participants identified as Muslim, all controls, too few to support inference. Hormonal treatment—far more frequent in patients (64.9% vs. 24.0%; *p* < 0.001), with no control on endometriosis-specific therapy or a GnRH analogue—likewise left the sofrito association (OR 0.28), the null overall adherence result and the lower body mass index (OR 0.90) unchanged. If anything, it masked the symptom burden, since the quality-of-life and sleepiness differences became stronger after adding it to the age-adjusted models (CVM-22 *p* = 0.048 to 0.005; ESS *p* = 0.062 to 0.008).

Across the 77 patients, anatomical severity was unrelated to every dietary, quality-of-life and sleep measure examined: PREDIMED-14 adherence (*p* = 0.27), sofrito consumption (*p* = 0.29), total CVM-22 score (*p* = 0.48), dysmenorrhoea (*p* = 0.58), constant pelvic pain (*p* = 0.19), body mass index (*p* = 0.12) and Epworth score (*p* = 0.97) did not differ across stages I to IV. Anatomical extent and symptom or dietary profile therefore appear largely independent, consistent with the well-documented dissociation between ASRM stage and symptom severity in endometriosis.

Finally, because item 19 of the CVM-22 is worded in the opposite direction to the other items, we repeated the total-score comparison excluding it (median 54 vs. 36; *p* = 0.004) and with it reverse-scored (median 54 vs. 38; *p* = 0.003); the difference reported in Table 1 and Table 3 was robust to both.

The item-level distribution also indicates limited discriminatory scope for this instrument in a Spanish population. Use of extra-virgin olive oil as the main culinary fat was universal (100% of participants in both groups), so this defining Mediterranean diet criterion carries no between-group variance at all, and several further items were met by a large majority (poultry preferred over red meat, 84.7%; sofrito, 74.0%; olive oil quantity, 67.2%). Adherence was not uniformly high, however: fruit (41.8%), legumes (43.5%) and fish (50.3%) intake fell well short of the PREDIMED targets, and although 70.1% reached the conventional adequate adherence threshold (≥8), only 33.9% scored ≥10 and 7.3% scored ≥12 out of 14 (mean 8.5 ± 2.1). The dietary profile of this cohort is therefore best described as a culturally shared Mediterranean culinary base with universal olive oil use, superimposed on only moderate attainment of the quantitative food group targets.

Individual ESS item scores are shown in Table 4; only one of eight items (dozing while sitting and talking to someone) reached significance after false discovery rate correction, so the item-level evidence for a sleep difference is limited. Overall, physical activity showed only a non-significant trend and overall Mediterranean diet adherence did not distinguish women with endometriosis from controls; body mass index was slightly lower in cases, daytime sleepiness was marginally higher but not robust to age adjustment, and dietary differences were confined to a single item (lower sofrito consumption in cases).

### 3.3. Exploratory Machine Learning Discrimination

**Figure 1:**
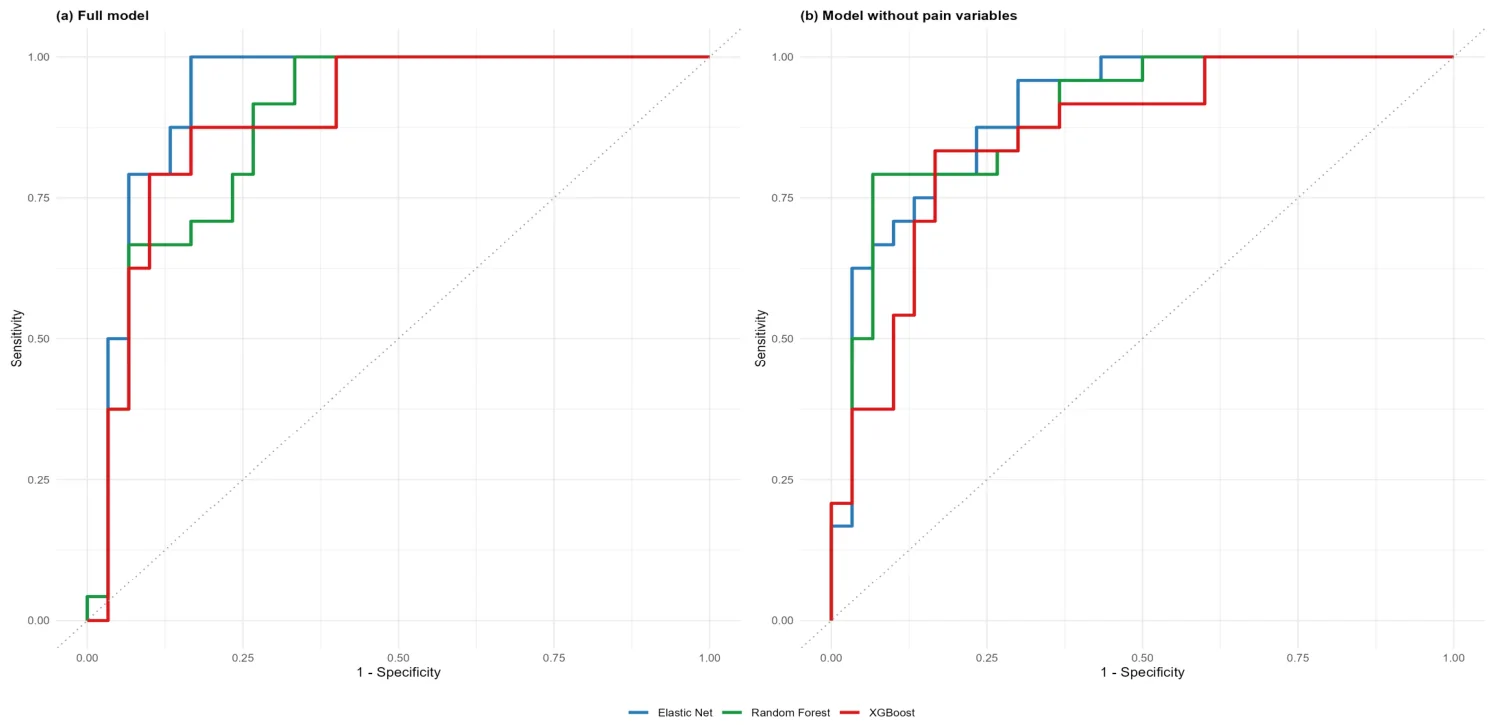
ROC curves on the held-out test set. (**a**) Full model. (**b**) Model without pain variables. AUC-ROC with 95% CIs in Supplementary Table S3; dashed diagonal = no discrimination.

This secondary analysis asked how far self-reported data separate diagnosed cases from controls, and what drives that separation. Table 5 presents the three algorithms for the full model and for a model without the pain scales (ROC curves in Figure 1). Discrimination was high in the full model, with Elastic Net performing best (AUC-ROC = 0.933, 95% CI 0.851–0.993), with XGBoost (0.890) and Random Forest (0.882) close behind; the test set contained only 24 cases, so threshold-based estimates such as the 100% sensitivity are fragile. Cross-validated AUC-ROC (0.946, SE 0.010) agreed closely with the held-out value. Excluding the pain scales cost little (Elastic Net AUC-ROC = 0.903), because the CVM-22 quality-of-life items—themselves consequences of the disease—still carry substantial symptom information; this pain-free model was, however, miscalibrated, and its cross-validated estimate (0.780) was markedly lower than its test value. Calibration curves, bootstrap confidence intervals, and Precision–Recall curves are given in Supplementary Figures S2 and S3, and Table S3.

**Figure 2:**
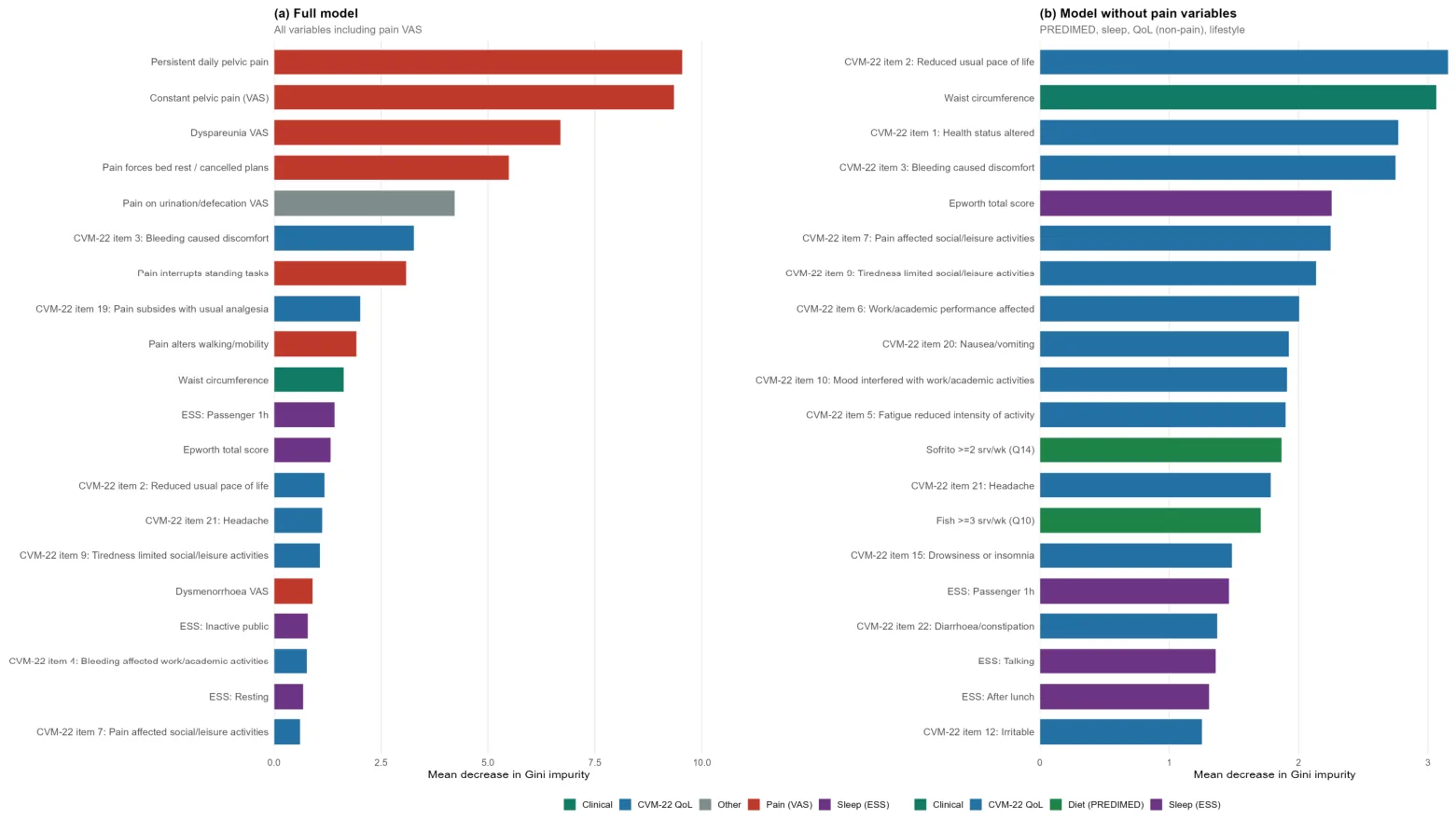
Random Forest variable importance. (**a**) Full model. (**b**) Model without the pain VAS (CVM-22 items retained). Ranked by mean decrease in Gini impurity; CVM-22 items labelled by scale number. Colours = variable domain.

Because the full feature set is large relative to the number of events, we also fitted a parsimonious unpenalised logistic model whose size respects the sample-size limit for prediction models [28], with seven predictors (constant pelvic pain, dyspareunia, pain on urination or defecation, bleeding-related discomfort (CVM-22 item 3), need for bed rest, waist circumference, and first-degree family history). It discriminated almost as well as the full model (cross-validated AUC-ROC 0.903; held-out 0.854), but was less well calibrated (slope 0.45), so the discrimination does not depend on the large feature set. Variable importance (Figure 2) showed the same picture: the leading predictors were pain measures and CVM-22 quality-of-life items, waist circumference (rank 10) being the only non-symptom variable among the top predictors of the full Random Forest model, and even without the pain scales, dietary and sleep variables featured only modestly (sofrito rank 12, fish rank 14, Epworth items in the lower half). Discrimination therefore rests on symptom-driven quality-of-life burden rather than on nutrition or lifestyle.

### 3.4. Risk Factor Analysis (Antecedent Variables)

The machine learning model in Section 3.3 discriminates diagnosed cases from controls but relies largely on symptom and quality-of-life variables that are consequences of established disease. To address the distinct question of risk factor association, we fitted logistic regression models restricted to variables that plausibly precede disease onset (first-degree family history, age at menarche, body mass index, and smoking), with age as a confounder (Table 6). We use ‘antecedent’ to mean that these characteristics are not themselves symptoms of established disease; two of them—body mass index and smoking—were nonetheless recorded at the survey rather than before diagnosis, so current body mass index is only a proxy for pre-disease body habitus and any antecedent reading of it must remain provisional (see Discussion). Because the design is case-control with an artificial case:control ratio, these models estimate relative associations (odds ratios), not absolute risk. Of the candidate factors, only a first-degree family history of endometriosis was robustly associated with case status, both unadjusted (OR = 7.95 (2.21–28.59)) and after adjustment for age (OR = 14.96 (3.41–65.66); *p* < 0.001), consistent with the well-documented heritability of the condition. Age at menarche and smoking showed no significant association. Body mass index was inversely associated with case status after age adjustment (OR = 0.86 (0.78–0.94)), an association that was borderline in the crude analysis (OR = 0.93 (0.87–1.00)). Because it emerges only once age is taken into account, and age is itself strongly related to case status here, it must be read with caution; however, it was not driven by outlying values, being confirmed by a median-based test, unaffected by winsorising and stronger rather than weaker when obese participants were excluded. Body mass index is, however, a crude anthropometric measure that does not distinguish fat from lean mass or capture fat distribution—visceral adiposity being more informative—so we do not attach a biological interpretation to this association. In an age-matched subsample (ages 25–35 years), only 18 cases and 18 controls were available, which was insufficient for stable estimation (family history perfectly separated the groups). These results indicate that, beyond heredity, the collected data do not support robust, age-independent risk factors, and that a valid absolute risk or prospective risk prediction study would require an age-matched or population-based cohort design.

Because body mass index was inversely associated with case status, we explored its association across World Health Organization categories (Supplementary Table S2). The lower BMI in cases appeared concentrated at the low extreme of the distribution; however, the underweight and obese cells contained only 6 and 16 women, respectively, and the corresponding age-adjusted odds ratio for the underweight category (OR = 10.66 (1.16–98.43)) rests on so few observations that its confidence interval is uninformatively wide. We therefore do not interpret this category-level estimate substantively; it is reported only to indicate the direction of the effect and as a hypothesis for adequately powered studies. The direction of the overall association (Table 6) is nonetheless consistent with prior reports linking endometriosis to a leaner body habitus [33].

To quantify directly how much of the discrimination is attributable to pre-disease and nutritional factors rather than to established symptoms, we trained an Elastic Net model restricted to antecedent and dietary variables only (first-degree family history, age at menarche, body mass index, smoking, PREDIMED-14 total score, physical activity, and fish and sofrito intake). Its discrimination was modest—cross-validated AUC-ROC = 0.67 (SE 0.02) and held-out test AUC-ROC = 0.73—far below the symptom-based models (test AUC-ROC 0.90–0.93). This quantifies the central message of the study: antecedent and nutritional variables carry only limited discriminatory information, and the high accuracy of the full model derives almost entirely from concurrent symptom and quality-of-life burden.

## 4. Discussion

This study asked primarily whether women with endometriosis differ from controls in lifestyle, dietary, and sleep profiles and, secondarily, what drives the discrimination of diagnosed endometriosis from self-reported data. Once cases and controls were of comparable age, what women ate and how much they exercised no longer separated the two groups; what did separate them was how much they were suffering. Body mass index was slightly lower in cases and daytime sleepiness marginally higher, the latter not robust to age adjustment, and the only dietary item to survive correction for multiple testing was sofrito. Pain and menstrual quality of life, by contrast, were substantially worse in cases even against age-comparable controls, indicating that this burden is not an artefact of comparing patients with younger women. The exploratory discrimination analysis reproduced the same asymmetry: the groups were separated on the basis of symptoms and their impact on daily life, not on nutrition or lifestyle (Table 5, Figure 2).

Physical activity showed only a weak trend toward lower levels in cases, and this disappeared once age was taken into account (Table 2). Chronic pelvic pain, dysmenorrhoea and fatigue are plausible reasons why some patients move less [3,19], but in a design such as ours physical activity cannot be regarded as a reliable marker of the disease. This does not diminish its therapeutic interest: exercise has been reported to reduce pain and improve quality of life in endometriosis [19], and that is a question for intervention trials rather than for a cross-sectional comparison.

The dietary findings were modest and confined to a single item. Overall adherence to the Mediterranean diet was practically identical in the two groups, whether measured by the total PREDIMED-14 score or by the proportion of women reaching the adequate adherence threshold (Table 2). Among the 13 testable individual items, only sofrito survived correction for multiple testing, and it was consumed less—not more—by the patients. Because our cases came from across Spain while most controls were Andalusian, we asked whether this simply reflected regional cooking habits. It did not: the difference barely moved when we adjusted for region, and it was still present when we compared Andalusian women only. Nor was it explained by the socioeconomic indicators available: household income did not differ between groups, sofrito intake was similar across income bands, adjustment for income left the association unchanged, and adjustment for religious or cultural identification attenuated it only slightly (Section 3.2). Socioeconomic position deserves attention because socioeconomic gradients influence both access to an endometriosis diagnosis and diet; our only indicator, however, was a coarse three-band income measure, and education was not recorded, so residual socioeconomic confounding cannot be excluded. The lower use of sofrito among patients was therefore robust to the measured confounders and to multiple testing, but it rests on a single item in a cross-sectional comparison and remains open to the unmeasured differences between recruitment routes discussed below.

Sofrito is the aromatic culinary base of much traditional Spanish and Mediterranean cooking and the only PREDIMED-14 item that captures culinary technique rather than the purchase of a food. Relative to controls, women with endometriosis reported less sofrito but nominally more fish and nuts (Table 2), a pattern compatible with a shift away from traditional, time-consuming home cooking towards individual foods publicised as anti-inflammatory after diagnosis. A disease-capacity explanation—pain and fatigue limiting slow, standing preparation—is not supported by our data: within the patient group sofrito intake was unrelated to pain, to CVM-22 burden or to ASRM stage (Kruskal-Wallis *p* = 0.29; below the control level at every stage), and symptom severity did not modify the case-control association (Section 3.2). The absence of any dose-response leaves post-diagnosis dietary change as the more plausible reading, although the cross-sectional design cannot establish it. We therefore make no causal claim: whether lower sofrito intake contributes to endometriosis is a question these data cannot address, and reverse causation is the more likely direction.

The recruitment asymmetry must be weighed critically in interpreting this finding. Cases were members of a patient association, already diagnosed and engaged with their health; they are likely to have higher disease-specific health literacy, greater contact with healthcare and with dietary advice (often circulated within patient communities), and may have modified their diet after diagnosis or report it differently from women recruited in community settings. Any of these could lower the reported frequency of a traditional preparation such as sofrito independently of the disease itself, and the different geographic and cultural composition of the groups, although adjusted for, adds scope for residual confounding. The finding should therefore be regarded strictly as hypothesis-generating. An independent validation would require (i) a different, ideally population-based or consecutively recruited clinical sample with diagnoses verified against surgical or imaging records; (ii) assessment of diet with a validated food frequency instrument that captures culinary techniques, not only foods; and (iii) information on the timing of dietary changes relative to symptom onset and diagnosis—ideally by recruiting women at referral, before dietary counselling—so that post-diagnosis change can be separated from pre-existing habits. Only if replicated under these conditions would supporting patients to maintain feasible home cooking become a defensible dietary message.

Microbiota-mediated pathways, including the estrobolome, have been proposed to link diet with the estrogen-dependent biology of endometriosis, and gut dysbiosis has been described in affected women [34,35]. Because the microbiome was not measured here and no study has examined sofrito in relation to endometriosis, we do not interpret our finding in mechanistic terms; such pathways can only be addressed by future studies that combine dietary assessment with microbiome biomarkers.

The absence of an overall dietary difference should also be read in the light of what this population eats. Extra-virgin olive oil was the main cooking fat for every participant, and most preferred poultry to red meat and used sofrito regularly (Table 2); where a habit is universal, it cannot distinguish two groups, so part of the apparent null reflects how little variation exists in the shared Mediterranean base of a Spanish sample rather than the absence of any diet–disease relationship. This also limits generalisation: the sample offers almost no contrast on olive oil, the very exposure that most distinguishes the Mediterranean diet internationally, and more variably eating populations may be better placed to detect associations. It would nonetheless be wrong to infer that these women have little room for improvement: adherence was moderate rather than high, with fewer than half reaching the recommended intake of fruit or legumes (Table 2), and it is on those quantitative goals that intervention could still act.

Fish was eaten more often by cases, and this became statistically significant once age was taken into account, but it did not survive correction for multiple testing and should be treated as no more than a hypothesis. Long-chain omega-3 fatty acids from fish have been proposed to modulate prostaglandin synthesis and pelvic inflammation [13], but a deliberate increase in fish intake after diagnosis is at least as likely an explanation. Overall, and in contrast to reviews suggesting that Mediterranean diet components may attenuate endometriosis severity [13], our data do not support a disease-specific dietary signature.

The sleep findings were modest. Daytime sleepiness was marginally higher in cases in the unadjusted comparison, but the difference was not significant after age adjustment, and only one of the eight Epworth items survived correction (Table 4); these data therefore do not establish a distinct sleep phenotype in endometriosis, although they are directionally consistent with reports of impaired sleep in the disease [20]. A mechanism relevant to the pain burden is central sensitisation, an amplification of nociceptive processing in the central nervous system that affects a substantial proportion of women with endometriosis and is associated with more severe and more persistent pain and with poorer response to hormonal treatment [36,37,38]. Poor sleep quality is also common in endometriosis and is related to pain symptoms [39], so central sensitisation, pain and sleep disturbance are likely to be interrelated. Central sensitisation was not assessed here, for example with the Central Sensitization Inventory, and may partly explain why pain and quality-of-life impairment were unrelated to ASRM stage; future studies should measure it together with objective sleep indicators such as actigraphy or polysomnography.

The machine learning analysis must be interpreted within clear limits. Its dominant predictors—constant and persistent pelvic pain, dyspareunia, pain on urination or defecation, need for bed rest and the CVM-22 item on bleeding-related discomfort—are concurrent manifestations of established disease, not antecedent risk factors. Cases were already diagnosed and symptomatic, while controls were disease-free, so the model discriminates established, symptomatic disease from health; it does not predict future risk, it is not a screening or triage tool, and it has not been externally validated. Because the diagnosis was self-reported, any misclassification would also propagate into the model. Its relevance to the nutritional question is a quantitative, essentially negative one: restricted to antecedent and nutritional variables, discrimination fell to a cross-validated AUC-ROC of 0.67 (test 0.73), showing that the separability of cases from controls owes little to diet or lifestyle and almost everything to established symptoms.

When we set the symptoms aside and looked only at characteristics that precede the disease, two stood out (Table 6). A first-degree family history was by far the strongest correlate, consistent with the well-established heritable component of endometriosis. A lower body mass index was also associated after age adjustment, but this finding should be interpreted with considerable caution: it emerges only once age is accounted for, body mass index was measured at the survey rather than before diagnosis, and it is a crude anthropometric index that does not distinguish fat from lean mass or capture fat distribution, for which visceral adiposity is more informative. The direction is consistent with prospective evidence linking a leaner body habitus to a higher likelihood of endometriosis [33], but pain, disease burden or hormonal treatment may themselves affect weight, so no causal or biological reading is justified. No dietary, sleep or behavioural variable emerged as an independent antecedent factor, and quantifying genuine risk would require a prospective, population-based cohort rather than a case-control sample such as ours.

Previous ML-based endometriosis studies have mostly used biochemical markers or imaging findings [21,22]. Because our controls were of comparable reproductive age, our discrimination estimate is less inflated by demographic contrast than a young control comparison would be, but it still reflects a diagnosed-versus-disease-free contrast rather than a primary care triage scenario.

### 4.1. Limitations

Several limitations warrant consideration. First, and most importantly, the diagnosis, its confirmatory procedure, and the ASRM stage were self-reported and were not verified against clinical or surgical records. Women were required to name the confirmatory procedure and were recruited through a patient association whose members have usually undergone a documented diagnostic process, but misclassification of case status or stage cannot be excluded. Misclassified cases would bias the group comparisons towards the null and would propagate into the discrimination analysis, and self-reported stage may explain part of its weak relationship with symptoms.

Second, the two groups were reached in different ways—patients through a national patient association, controls consecutively from the general population of a single region—which creates a substantial risk of selection bias and residual confounding. The groups differed in geographic origin and cultural identification, and probably also in unmeasured characteristics such as health awareness, health literacy, healthcare engagement and exposure to dietary advice; patients may have changed their diet after diagnosis or report it differently. We adjusted for age, region, income, cultural identification, and hormonal treatment, but adjustment cannot remove unmeasured differences, and this asymmetry also limits how far the findings extend to undiagnosed women presenting in primary care. Residual confounding by age may also persist despite age adjustment, because patients remained somewhat older.

Third, endometriosis was treated as a single condition. Information on lesion phenotype (ovarian endometrioma, superficial peritoneal disease, deep infiltrating endometriosis, adenomyosis) and on bowel involvement was not collected, and only a self-reported ASRM stage was available. Because these phenotypes differ in pain profile and, potentially, in their relationship with diet and gut-related pathways, pooling them may have introduced heterogeneity and obscured phenotype-specific associations; previous surgery, including the two bowel resections, was reported only as free text, although excluding the latter did not change the results.

Fourth, dietary adherence was captured only by the PREDIMED-14, a brief screener of largely dichotomous items designed to grade overall Mediterranean diet adherence rather than to quantify intake. It does not measure food quantities, nutrient intakes, or dietary patterns beyond its fourteen items, one of which (olive oil as the main fat) carried no variance at all in this sample. The absence of an overall dietary difference is therefore conditional on a coarse instrument: a genuine difference in dietary quantity or composition cannot be excluded, and a validated food frequency questionnaire or weighed dietary record would be needed to settle the question.

Fifth, the sample is modest for the number of candidate predictors in the discrimination analysis, which exceeds the recommended sample-size limit [28]. Penalisation, repeated cross-validation and a parsimonious model that respects that limit (cross-validated AUC-ROC 0.903 versus 0.946) indicate that the discrimination is not an artefact of overfitting, but some optimism cannot be excluded and no external validation was performed. Finally, the study is cross-sectional and relies on self-reported questionnaires, so it cannot establish temporality or causation and is open to recall bias, and the sample, although geographically varied, cannot support claims of transcultural validity.

### 4.2. Strengths

The study also has clear strengths. The most important is the control group: by deliberately recruiting women of comparable reproductive age, we removed the age confounding that undermines most case-control studies of lifestyle in endometriosis, and this is what allows the dietary null result to be interpreted at all. We corrected for multiple comparisons throughout, which distinguishes robust item-level findings from chance, used validated instruments (PREDIMED-14, IPAQ, ESS, CVM-22 and visual analogue scales) rather than ad hoc questions, and tested the main dietary finding against the measured confounders, previous surgery and symptom severity. The machine learning was conducted with leakage-free preprocessing, repeated cross-validation and calibration assessment, and we report openly that its accuracy rests on symptoms rather than on nutrition.

## 5. Conclusions

The principal nutritional message of this study is a well-controlled null result: in an age-comparable case-control sample with correction for age and multiple testing, overall Mediterranean diet adherence and physical activity did not distinguish women with endometriosis from controls. This tempers earlier, age-confounded reports of strong protective dietary associations and argues against a disease-specific dietary signature detectable by these instruments, within the limits of a brief dietary screener and of self-reported diagnoses. It also gives no support to restrictive ‘anti-inflammatory’ diets, which lack backing from high-quality trials [16]. Body mass index was modestly lower in the patients, and daytime sleepiness was marginally higher but not robustly so after age adjustment.

Sofrito, the only PREDIMED-14 item to survive correction, was reported less often by women with endometriosis. Because this is a single-item, cross-sectional finding in groups recruited through different routes, and post-diagnosis dietary change is a plausible explanation, it should be regarded as hypothesis-generating; it requires validation in an independent sample with verified diagnoses, a dietary instrument that captures culinary technique, and information on the timing of dietary change relative to diagnosis.

In the exploratory discrimination analysis, self-reported data separated diagnosed endometriosis from controls of comparable age with high apparent accuracy (best AUC-ROC = 0.933), but this reflected concurrent symptom and quality-of-life burden: restricted to antecedent and nutritional variables, discrimination reached only AUC-ROC 0.67 (cross-validated) to 0.73 (test). The model is a proof-of-concept that discriminates established disease from health, not a risk prediction, triage, or early-detection tool. Among characteristics that precede the disease, only a first-degree family history was robustly associated with case status.

## Figures and Tables

**Table 1:** Participant characteristics by study group.

Variable	Total (*n* = 177)	Endometriosis (*n* = 77)	Control (*n* = 100)	*p*-Value
**Sociodemographic and anthropometric characteristics**				
Age (years)	34.3 ± 8.5	37.7 ± 7.6	31.7 ± 8.2	**<0.001**
Body mass index (kg/m^2^)	24.5 ± 4.7	23.7 ± 4.7	25.1 ± 4.6	0.049
Waist circumference (cm)	79.1 ± 15.7	77.5 ± 16.9	80.4 ± 14.7	0.232
Current smoker	20 (11.3%)	7 (9.1%)	13 (13.0%)	0.478
**Pain intensity—Visual Analogue Scale (VAS; 0 = no pain, 10 = maximum pain)**				
Dysmenorrhoea	5.0 [1.0–7.5]	6.2 [0.0–8.0]	4.0 [1.5–7.0]	0.098
Pain on urination/defecation (VAS)	0.0 [0.0–5.0]	5.0 [1.0–7.0]	0.0 [0.0–1.2]	**<0.001**
Dyspareunia	1.0 [0.0–5.0]	5.0 [2.0–7.0]	0.0 [0.0–2.0]	**<0.001**
Constant pelvic pain	2.0 [0.0–6.0]	5.0 [3.0–8.0]	0.0 [0.0–2.0]	**<0.001**
**Pain-related functional impairment (0 = never, 4 = almost always)**				
Persistent daily pelvic pain	2.0 [1.0–3.0]	3.0 [2.0–4.0]	1.0 [0.0–2.0]	**<0.001**
Pain interrupts tasks requiring standing	2.0 [1.0–3.0]	2.0 [2.0–3.0]	1.0 [0.0–2.0]	**<0.001**
Pain alters walking or mobility	1.0 [0.0–2.0]	2.0 [1.0–3.0]	1.0 [0.0–2.0]	**<0.001**
Pain forces bed rest/cancelled plans	2.0 [1.0–3.0]	3.0 [2.0–3.0]	1.0 [0.0–2.0]	**<0.001**
**Family history and menstrual profile**				
First-degree family history of endometriosis	19 (10.7%)	16 (20.8%)	3 (3.0%)	**<0.001**
Age at menarche (years)	12.0 ± 1.7	12.1 ± 1.8	12.0 ± 1.7	0.578
**Quality of life—CVM-22 summary scores**				
CVM-22 total score (range 0–66)	43.0 [26.0–59.0]	57.0 [16.0–63.0]	39.0 [26.0–53.0]	**0.006**
**Hormonal treatment and gynaecological history**				
Current hormonal therapy	*n* = 177	—	—	**<0.001**
—0. None	103 (58.2%)	27 (35.1%)	76 (76.0%)	—
—1. Combined contraceptive	23 (13.0%)	10 (13.0%)	13 (13.0%)	—
—2. Progestogen-only	8 (4.5%)	5 (6.5%)	3 (3.0%)	—
—3. Hormonal IUD	8 (4.5%)	5 (6.5%)	3 (3.0%)	—
—4. Endometriosis-specific	19 (10.7%)	19 (24.7%)	0 (0.0%)	—
—5. GnRH analogue/antagonist	5 (2.8%)	5 (6.5%)	0 (0.0%)	—
—6. Other/unspecified	11 (6.2%)	6 (7.8%)	5 (5.0%)	—
Previous surgery	51 (28.8%)	41 (53.2%)	10 (10.0%)	**<0.001**
—Laparoscopic excision	22 (12.4%)	20 (26.0%)	2 (2.0%)	—
—Hysterectomy	9 (5.1%)	9 (11.7%)	0 (0.0%)	—
—Laparotomy (open, unspecified)	4 (2.3%)	4 (5.2%)	0 (0.0%)	—
—Bowel resection	2 (1.1%)	2 (2.6%)	0 (0.0%)	—
—Other gynaecological	14 (7.9%)	6 (7.8%)	8 (8.0%)	—
ASRM stage (cases only)	*n* = 77	—	n/a	—
**Stage I**		**18 (23.4%)**	**n/a**	
**Stage II**		**16 (20.8%)**	**n/a**	
**Stage III**		**11 (14.3%)**	**n/a**	
**Stage IV**		**32 (41.6%)**	**n/a**	

*n* = 177 (77 endometriosis, 100 controls). Mean ± SD (*t*-test); median [IQR] (Mann–Whitney U); *n* (%) (chi-squared or Fisher). Bold: *p* < 0.05.

**Table 2:** Lifestyle, dietary and sleep characteristics by study group.

Variable	Total (*n* = 177)	Endometriosis (*n* = 77)	Control (*n* = 100)	*p*-Value	q (FDR)
**Physical activity—International Physical Activity Questionnaire (IPAQ)**					
Physical activity level	*n* = 177	—	—	0.082	
—Low or inactive	58 (32.8%)	29 (37.7%)	29 (29.0%)	—	
—Moderate	94 (53.1%)	42 (54.5%)	52 (52.0%)	—	
—High	25 (14.1%)	6 (7.8%)	19 (19.0%)	—	
**Mediterranean diet adherence—PREDIMED-14 questionnaire (0 = low, 14 = maximum adherence)**					
Total PREDIMED score (range 0–14)	8.5 ± 2.1	8.6 ± 2.2	8.5 ± 2.0	0.823	
Adequate adherence (score ≥ 8)	124 (70.1%)	54 (70.1%)	70 (70.0%)	0.985	
Q1: Olive oil as main culinary fat	177 (100.0%)	77 (100.0%)	100 (100.0%)	—	—
Q2: ≥4 tablespoons of olive oil/day	119 (67.2%)	52 (67.5%)	67 (67.0%)	0.940	0.940
Q3: ≥2 servings of vegetables/day	127 (71.8%)	53 (68.8%)	74 (74.0%)	0.449	0.940
Q4: ≥3 servings of fruit/day	74 (41.8%)	31 (40.3%)	43 (43.0%)	0.714	0.940
Q5: Avoidance of red/processed meat	125 (70.6%)	53 (68.8%)	72 (72.0%)	0.646	0.940
Q6: Avoidance of butter/cream	115 (65.0%)	55 (71.4%)	60 (60.0%)	0.114	0.371
Q7: Avoidance of sugar-sweetened beverages	118 (66.7%)	52 (67.5%)	66 (66.0%)	0.830	0.940
Q8: Wine consumption (≥7 glasses/week)	8 (4.5%)	3 (3.9%)	5 (5.0%)	0.726	0.940
Q9: ≥3 servings of legumes/week	77 (43.5%)	33 (42.9%)	44 (44.0%)	0.879	0.940
Q10: ≥3 servings of fish or seafood/week	89 (50.3%)	46 (59.7%)	43 (43.0%)	0.027	0.166
Q11: Avoidance of commercial sweets	103 (58.2%)	44 (57.1%)	59 (59.0%)	0.804	0.940
Q12: ≥3 servings of nuts/week	97 (54.8%)	49 (63.6%)	48 (48.0%)	0.038	0.166
Q13: White meat preference over red meat	150 (84.7%)	64 (83.1%)	86 (86.0%)	0.597	0.940
Q14: ≥2 servings of sofrito/week	131 (74.0%)	48 (62.3%)	83 (83.0%)	0.002	**0.025**
**Daytime sleepiness—Epworth Sleepiness Scale (ESS; range 0–24; ≥10 = excessive sleepiness)**					
ESS total score	9.5 ± 5.1	10.4 ± 5.6	8.8 ± 4.6	0.039	

*n* = 177. Totals: mean ± SD (*t*-test); physical activity: Fisher’s exact test; items: *n* meeting criterion (%) (chi-squared or Fisher). q = Benjamini–Hochberg-adjusted *p* across the 13 testable PREDIMED items (Q1 was met by all participants and is not testable); bold: q < 0.05. Age-adjusted analyses in text.

**Table 3:** CVM-22 quality-of-life item and total scores by group.

Subscale	CVM-22 Item (Scored 0–3)	Total (*n* = 177)	Endometriosis (*n* = 77)	Control (*n* = 100)	*p*-Value	q (FDR)
Health perception, physical & functional well-being	Item 1—My health status has been altered	2.0 [1.0–3.0]	3.0 [1.0–3.0]	1.0 [1.0–2.0]	<0.001	**0.0087**
	Item 2—Menstruation forced me to reduce my usual pace of life	1.0 [1.0–3.0]	3.0 [0.0–3.0]	1.0 [1.0–2.0]	0.001	**0.0088**
	Item 3—Bleeding has caused me discomfort	2.0 [1.0–3.0]	3.0 [0.0–3.0]	2.0 [1.0–3.0]	0.792	0.8712
	Item 4—Menstrual bleeding affected my work, academic or domestic activities	2.0 [1.0–3.0]	3.0 [0.0–3.0]	1.0 [1.0–2.2]	0.024	0.0534
	Item 5—Fatigue forced me to reduce the intensity of what I was doing	2.0 [1.0–3.0]	3.0 [1.0–3.0]	1.5 [1.0–3.0]	0.007	**0.0233**
	Item 6—My performance in work or academic activities was affected	2.0 [1.0–3.0]	3.0 [0.0–3.0]	1.0 [1.0–2.0]	0.007	**0.0233**
	Item 7—Pain affected my social and leisure activities	2.0 [1.0–3.0]	3.0 [0.0–3.0]	1.0 [1.0–2.0]	0.002	**0.0129**
	Item 8—Menstrual bleeding conditioned my social and leisure activities	2.0 [1.0–3.0]	2.0 [0.0–3.0]	1.0 [1.0–2.0]	0.051	0.0897
	Item 9—Tiredness limited my social and leisure activities	2.0 [1.0–3.0]	3.0 [0.0–3.0]	1.5 [1.0–3.0]	0.020	**0.0500**
Psychological & cognitive well-being	Item 10—My mood interfered with my work, academic or domestic activities	2.0 [1.0–3.0]	3.0 [1.0–3.0]	2.0 [1.0–3.0]	0.052	0.0897
	Item 11—I have felt sad	3.0 [1.0–3.0]	3.0 [1.0–3.0]	2.0 [1.0–3.0]	0.516	0.6676
	Item 12—I have been irritable	3.0 [1.0–3.0]	3.0 [1.0–3.0]	2.0 [2.0–3.0]	0.935	0.9346
	Item 13—I have had mood swings	3.0 [1.0–3.0]	3.0 [1.0–3.0]	3.0 [1.8–3.0]	0.894	0.9346
	Item 14—I have lacked concentration	2.0 [1.0–3.0]	3.0 [1.0–3.0]	2.0 [1.0–3.0]	0.003	**0.0141**
	Item 15—I have experienced drowsiness or insomnia	2.0 [1.0–3.0]	3.0 [0.0–3.0]	2.0 [1.0–3.0]	0.206	0.3024
	Item 16—I have had joint or muscular pain in the back and/or legs	2.0 [1.0–3.0]	3.0 [1.0–3.0]	2.0 [1.0–3.0]	0.105	0.1651
	Item 17—I have experienced frequent urination	2.0 [1.0–3.0]	2.0 [0.0–3.0]	1.0 [1.0–2.2]	0.053	0.0897
Symptoms	Item 18—Pain forced me to take medication	2.0 [1.0–3.0]	3.0 [0.0–3.0]	2.0 [1.0–3.0]	0.234	0.3212
	Item 19—Pain subsides with the usual analgesic treatment	2.0 [1.0–3.0]	2.0 [0.0–3.0]	3.0 [1.0–3.0]	<0.001	**0.0087**
	Item 20—I have experienced nausea and/or vomiting	1.0 [0.0–2.0]	1.0 [0.0–3.0]	1.0 [0.0–2.0]	0.020	**0.0500**
	Item 21—I have had a headache	2.0 [1.0–3.0]	2.0 [0.0–3.0]	2.0 [1.0–3.0]	0.765	0.8712
	Item 22—I have had diarrhoea or constipation	3.0 [1.0–3.0]	3.0 [1.0–3.0]	2.0 [1.0–3.0]	0.576	0.7040
Total score	TOTAL CVM-22 score (range 0–66)	43.0 [26.0–59.0]	57.0 [16.0–63.0]	39.0 [26.0–53.0]	0.006	—

Items scored 0 (not at all) to 3 (very frequent); higher = greater impact; labels abbreviated. Median [IQR]; Mann–Whitney U. q = FDR-adjusted *p* across the 22 items (total excluded); bold: q < 0.05 (items 9 and 20: q = 0.04996, shown as 0.0500).

**Table 4:** Epworth Sleepiness Scale item scores by group.

ESS Item (0–3)	Total (*n* = 177)	Endometriosis (*n* = 77)	Control (*n* = 100)	*p*-Value	q (FDR)
Item 1—Sitting and reading	1.0 [0.0–2.0]	1.0 [0.0–2.0]	1.0 [0.0–2.0]	0.638	0.638
Item 2—Watching television	2.0 [1.0–3.0]	2.0 [1.0–3.0]	2.0 [1.0–3.0]	0.555	0.635
Item 3—Sitting inactive in a public place (e.g., theatre or meeting)	0.0 [0.0–1.0]	0.0 [0.0–1.0]	0.0 [0.0–1.0]	0.065	0.183
Item 4—As a passenger in a car for one hour without a break	1.0 [0.0–2.0]	2.0 [1.0–2.0]	1.0 [0.0–2.0]	0.133	0.256
Item 5—Lying down to rest in the afternoon	2.0 [2.0–3.0]	3.0 [2.0–3.0]	2.0 [2.0–3.0]	0.069	0.183
Item 6—Sitting and talking to someone	0.0 [0.0–0.0]	0.0 [0.0–1.0]	0.0 [0.0–0.0]	<0.001	**<0.001**
Item 7—Sitting quietly after lunch, without alcohol	2.0 [1.0–3.0]	2.0 [1.0–3.0]	2.0 [1.0–2.2]	0.160	0.256
Item 8—In a car, stopped for a few minutes in traffic	0.0 [0.0–1.0]	0.0 [0.0–1.0]	0.0 [0.0–0.2]	0.332	0.443

Items scored 0 (no chance of dozing) to 3 (high chance). Median [IQR]; Mann–Whitney U. q = FDR-adjusted *p* across the 8 items. Bold: q < 0.05.

**Table 5:** Model performance on the held-out test set.

	Full Model (All Variables)			Model Without Pain Variables		
Metric	Elastic Net	Random Forest	XGBoost	Elastic Net	Random Forest	XGBoost
AUC-ROC	**0.933**	0.882	0.890	0.903	**0.896**	0.857
AUC-PR	**0.851**	0.798	0.779	0.863	**0.865**	0.817
Accuracy	**0.907**	0.796	0.852	0.778	**0.796**	0.778
Sensitivity	**1.000**	0.875	0.875	0.875	**0.792**	0.833
Specificity	**0.833**	0.733	0.833	0.700	**0.800**	0.733
PPV	**0.828**	0.724	0.808	0.700	**0.760**	0.714
NPV	**1.000**	0.880	0.893	0.875	**0.828**	0.846
F1-score	**0.906**	0.792	0.840	0.778	**0.776**	0.769
Cohen’s kappa	**0.816**	0.596	0.702	0.561	**0.589**	0.557
Balanced accuracy	**0.917**	0.804	0.854	0.787	**0.796**	0.783
Brier score	—	0.131	—	—	**0.145**	—

*n* = 54 (24 endometriosis, 30 controls). Metrics at the 0.5 threshold except AUC-ROC and AUC-PR. Bold: best model in each group.

**Table 6:** Odds ratios for antecedent (pre-disease) variables.

	Univariable		Multivariable	
Antecedent Factor	Crude OR (95% CI)	*p*	Age-Adjusted OR (95% CI)	*p*
First-degree family history (yes vs. no)	7.95 (2.21–28.59)	0.001	**14.96 (3.41–65.66)**	<0.001
Age at menarche (per year)	1.05 (0.88–1.25)	0.574	0.88 (0.70–1.11)	0.285
Body mass index (per kg/m^2^)	0.93 (0.87–1.00)	0.053	**0.86 (0.78–0.94)**	0.001
Current smoking (yes vs. no)	0.67 (0.25–1.77)	0.418	0.49 (0.14–1.76)	0.273
Age (per year)	1.09 (1.05–1.14)	<0.001	**1.14 (1.08–1.20)**	<0.001

Logistic regression, crude and age-adjusted, on *n* = 156 complete cases. Given the case-control design, ORs estimate relative association, not absolute risk. Bold: adjusted *p* < 0.05.

## Data Availability

The data presented in this study are available on reasonable request from the corresponding author. The data are not publicly available due to privacy restrictions.

## Supplementary Materials

The following supporting information can be downloaded at: https://www.mdpi.com/article/10.3390/nu18213629/s1, Methods S1, full machine learning pipeline specification (resampling seeds, imputation, SMOTE settings and tuning grids); Figure S1: geographic origin of participants by study group; Figure S2: Precision–Recall curves on the held-out test set; Figure S3: calibration of the Elastic Net and Random Forest models; Table S1: standardized Elastic Net coefficients of the best full model; Table S2: odds of endometriosis by World Health Organization body mass index category; Table S3: bootstrap 95% confidence intervals for AUC-ROC.
