# Supplementary material for: Mediterranean Diet, Physical Activity, and Sleep in Endometriosis: An Age-Comparable Case-Control Study with Exploratory Machine Learning Discrimination
Source: Nutrients. 2026 Oct 31;18(21):3629. doi: 10.3390/nu18213629 (PMC13764908; doi:10.3390/nu18213629)
Supplement: Supplementary file 1 [file nutrients-18-03629-s001.zip › nutrients-4484955-supplementary.pdf]

Supplementary Materials

Mediterranean Diet, Physical Activity, and Sleep in Endometriosis: An Age-Comparable Case-Control Study with Exploratory Machine Learning Discrimination

Methods S1. Machine-learning pipeline specification

This document reports the complete specification of the discrimination models summarised in Section 2.4 of the main text, in sufficient detail for exact reproduction. All analyses were run in R 4.6.0 with the tidymodels framework (version 1.5.0); the engines were glmnet (Elastic Net), ranger (Random Forest) and xgboost (XGBoost), with SMOTE provided by themis. Every random seed used is stated below, so that re-running the pipeline reproduces the reported figures exactly.

S1.1. Data partition and resampling

The analysed sample (n = 177; 77 cases, 100 controls) was divided into training and test sets by stratified random sampling on case status, with 70% allocated to training (n = 123) and 30% held out (n = 54; 24 cases, 30 controls). The partition was generated with seed 123 and the identical seed was used for the full model and for the model without pain variables, so that the two share the same training and test membership and their performance is directly comparable. Hyperparameters were tuned by repeated stratified cross-validation within the training set only: 10 folds, 5 repeats (50 resamples in total).

S1.2. Preprocessing

Preprocessing was specified as a recipe and therefore estimated separately within the analysis portion of each resample, never on the full training set and never on the test set. This applies in particular to imputation, normalisation and SMOTE, and is what prevents information leaking from held-out data into the fitted models. The steps were applied in the following order: median imputation of numeric predictors; modal imputation of nominal predictors; assignment of previously unseen factor levels to a 'new' category; conversion of nominal predictors to dummy variables; removal of zero-variance predictors; normalisation of numeric predictors to mean zero and unit variance; and SMOTE oversampling of the minority class to parity (over_ratio = 1). Normalisation was applied only for the Elastic Net, since tree-based models are invariant to monotone rescaling of predictors; the Random Forest and XGBoost recipes were otherwise identical. Missing data were sparse: no predictor exceeded 5% missingness.

S1.3. Algorithms and tuning grids

Three algorithms were tuned over the grids below. Grid points were evaluated on all 50 resamples, and the configuration maximising the area under the precision-recall curve (AUC-PR) was carried forward and fitted once to the full training set before evaluation on the untouched test set. AUC-PR was preferred to AUC-ROC as the selection criterion because it is the more informative summary when the positive class is the minority.

Algorithm	Hyperparameter	Range or value	Grid	Seed
Elastic Net	penalty	10^-4 to 10^0	regular, 15 x 5 = 75 points	456
	mixture	0 to 1 (ridge to lasso)		

Algorithm	Hyperparameter	Range or value	Grid	Seed
Random Forest	mtry	3 to 15	regular, 4 x 4 = 16 points	789
	min_n	2 to 10		
	trees	1000 (fixed)		
XGBoost	tree_depth	2 to 6	space-filling, 20 points	321
	min_n	2 to 15		
	learn_rate	10^-3 to 10^-0.5		
	loss_reduction	10^-3 to 10^1		
	trees	500 (fixed)		
	sample_size	0.8 (fixed)		

Random Forest variable importance was computed as mean decrease in Gini impurity (ranger importance = 'impurity'). Blank cells repeat the algorithm, grid or seed of the row above.

S1.4. Selected hyperparameters

The configurations selected by the procedure above were as follows.

Model	Setting
Full model - Elastic Net	penalty = 1e-04; mixture = 0
Full model - Random Forest	mtry = 11; min_n = 4
Full model - XGBoost	min_n = 2; tree_depth = 4; learn_rate = 0.1274; loss_reduction = 0.5456
Without pain - Elastic Net	penalty = 0.07197; mixture = 0
Without pain - Random Forest	mtry = 3; min_n = 4
Without pain - XGBoost	min_n = 3; tree_depth = 5; learn_rate = 0.001833; loss_reduction = 1.438

Tuned parameters only; fixed parameters are given in Section S1.3.

One feature of these results is worth making explicit. In both models the Elastic Net mixing parameter was tuned to zero, that is, to a pure ridge penalty. Ridge regression shrinks coefficients towards zero but never to exactly zero, so the selected model retains all candidate predictors and performs no variable selection. The standardized coefficients in Table S1 therefore rank predictors by magnitude but should not be read as identifying a reduced subset, and the same caution applies to the Random Forest importances in Figure 3. That cross-validation preferred ridge over any sparser alternative is itself informative: discrimination rests on many correlated symptom and quality-of-life items acting together rather than on a small number of separable markers, which is consistent with the correlated structure of the CVM-22 and pain scales.

S1.5. Evaluation

Discrimination on the held-out set was summarised by AUC-ROC and AUC-PR, with 95% confidence intervals obtained by the percentile method from 1000 bootstrap resamples of the test-set predictions (seed 2024; Table S3). Threshold-based measures (accuracy, sensitivity, specificity, predictive values, F1, Cohen's kappa, balanced accuracy) were computed at a 0.5 cut-off, and overall probabilistic accuracy by the Brier score. To

expose any optimism arising from a single partition, the cross-validated AUC-ROC of the selected Elastic Net configuration was extracted from the same 50 resamples used for tuning and is reported alongside the held-out value in Section 3.3.
Calibration was quantified by the calibration slope and intercept. Writing p for the predicted probability and y for observed case status, the slope is the coefficient of the logit of p in a logistic regression of y on that logit, and the intercept is the constant from a logistic regression of y on the logit of p entered as an offset. A slope of one with an intercept of zero denotes perfect calibration; slopes above one indicate predicted probabilities compressed towards the middle of the range. The calibration plots (Figure 2) group predictions into six equal-width probability bins.

S1.6. Antecedent-only model

The model restricted to antecedent and nutritional variables reported in Section 3.3 used the same partition seed, the same recipe and the same Elastic Net grid as above. It differs in one respect, stated here for transparency: because that model is not intended for classification at a threshold but as an estimate of how much discrimination is attributable to pre-disease and dietary information, its hyperparameters were selected on AUC-ROC rather than AUC-PR. Its performance was AUC-ROC = 0.67 cross-validated and 0.73 on the held-out split.

S1.7. Code availability

The analysis is organised as a single reproducible pipeline: data cleaning and derivation of all study variables, model fitting and evaluation, and generation of every figure and table reported in the manuscript. The scripts are available from the corresponding author on reasonable request.

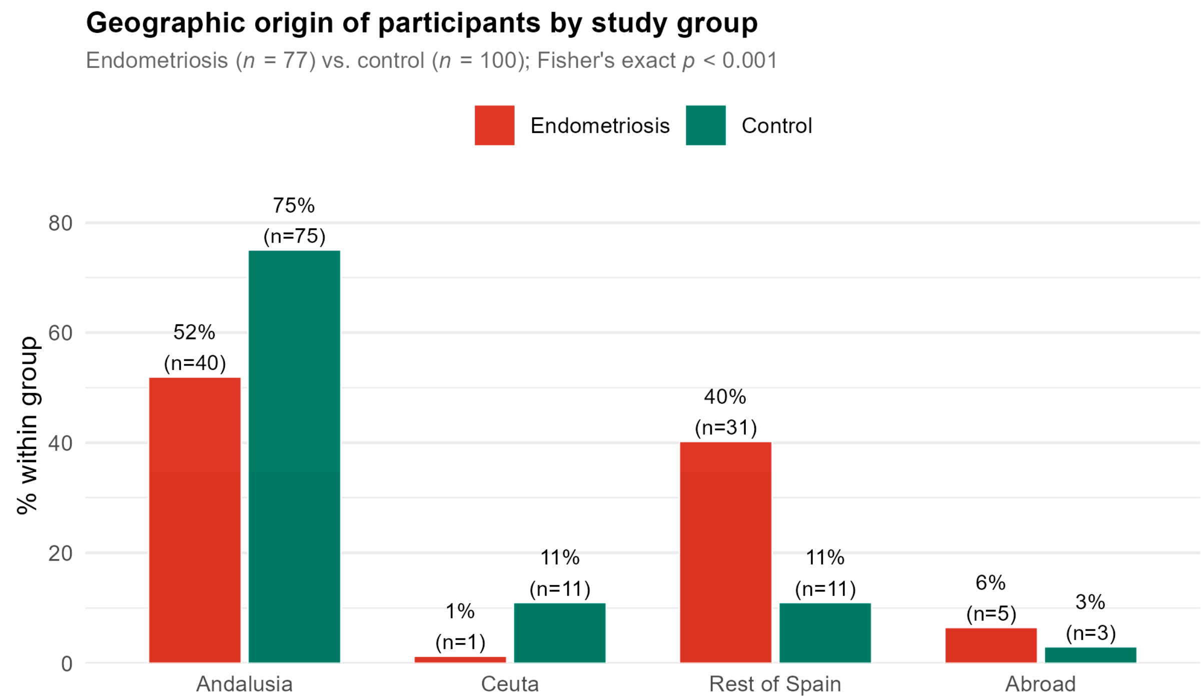

Figure S1. Geographic origin of participants by study group ($n = 177$). Fisher's exact test $p < 0.001$. The difference reflects the recruitment design, not a geographic risk factor.

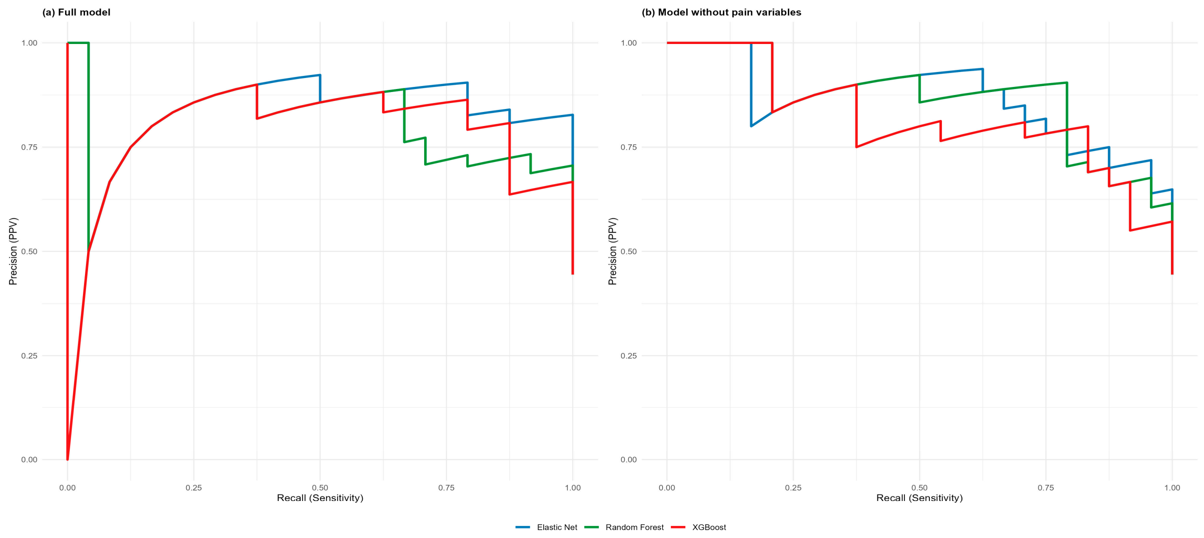

Figure S2. Precision–Recall curves on the held-out test set. (a) Full model (best AUC-PR: Elastic Net, 0.851). (b) Model without pain variables (best: Random Forest, 0.865). The horizontal reference is the no-skill baseline (prevalence = 43.5%).

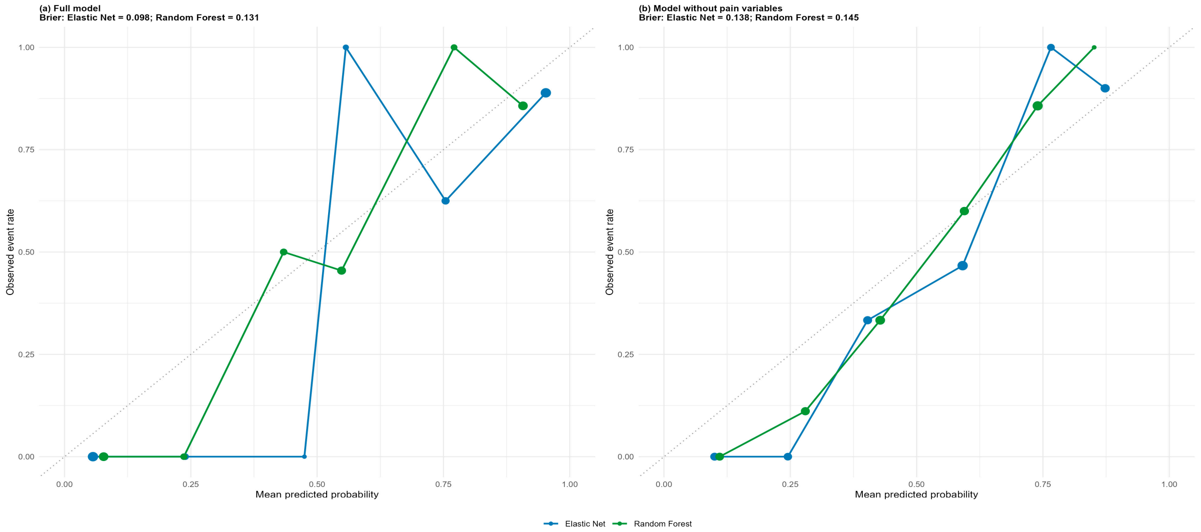

Figure S3. Calibration of the Elastic Net and Random Forest models. (a) Full model (Elastic Net slope 0.79, intercept −0.75). (b) Model without pain variables. Observed event rate versus mean predicted probability within six equal-width probability bins; point size is proportional to bin count; the diagonal denotes perfect calibration; Brier scores are given in each panel.

Table S1. Standardized Elastic Net coefficients of the best full model.

Predictor	Std. Coefficient
Persistent daily pelvic pain (frequency)	−0.81
Constant pelvic pain (VAS)	−0.71
Pain forces bed rest/cancelled plans (frequency)	−0.59
CVM-22 item 19	+0.55
Dyspareunia (VAS)	−0.53
Pain on urination/defecation (VAS)	−0.51
CVM-22 item 21	+0.50
CVM-22 item 3	+0.46
Family history of endometriosis: unknown (vs. no)	+0.41
CVM-22 item 12	+0.38
CVM-22 item 14	−0.35
Family history of endometriosis: yes (vs. no)	−0.34
CVM-22 item 1	−0.32
CVM-22 item 2	−0.31
CVM-22 item 8	−0.31
Waist circumference	+0.30
Pain interrupts tasks requiring standing (frequency)	−0.29
CVM-22 item 13	+0.26
CVM-22 item 5	−0.23
CVM-22 item 20	+0.21

Normalized predictors; 20 largest by absolute value of the 44 model terms. Tuning selected a pure ridge penalty (mixture = 0), so no coefficient is shrunk exactly to zero and the model performs no variable selection; magnitudes rank predictors but do not exclude any. Signs follow the model's internal coding and should be read alongside Tables 2–4 of the main text. VAS, visual analogue scale.

Table S2. Odds of endometriosis by WHO body mass index category.

BMI Category (WHO)	*n*	Endometriosis, n (%)	Age-Adjusted OR (95% CI)	*p*
Underweight (<18.5)	6	5 (83%)	**10.66 (1.16–98.43)**	**0.037**
Normal weight (18.5–25)	107	49 (46%)	1.00 (reference)	—
Overweight (25–30)	46	15 (33%)	0.57 (0.26–1.23)	0.150
Obesity (≥30)	18	8 (44%)	0.67 (0.22–2.01)	0.474

n = 177; age-adjusted logistic regression, reference = normal weight. Underweight and obese cells are small and their confidence intervals correspondingly wide. Bold: $p < 0.05$. BMI, body mass index (kg/m^2); WHO, World Health Organization.

Table S3. Bootstrap 95% confidence intervals for AUC-ROC.

	**Full Model**		**Model Without Pain**	
**Model**	**AUC-ROC**	**95% CI**	**AUC-ROC**	**95% CI**
Elastic Net	**0.933**	**0.851–0.993**	**0.903**	**0.808–0.968**
Random Forest	0.882	0.785–0.961	0.896	0.803–0.968
XGBoost	0.890	0.794–0.969	0.857	0.748–0.944

1000 resamples, percentile method. Bold: best model in each group.
